# Antitumour effects of PLC-*γ*1-(SH2)_2_-TAT fusion proteins on EGFR/c-erbB-2-positive breast cancer cells

**DOI:** 10.1038/sj.bjc.6601506

**Published:** 2004-01-06

**Authors:** Y Katterle, B H Brandt, S F Dowdy, B Niggemann, K S Zänker, T Dittmar

**Affiliations:** 1 Institute of Immunology, University of Witten/Herdecke, Stockumer Strasse 10, 58448 Witten, Germany; 2 Institute for Clinical Chemistry and Laboratory Medicine, Westfälische-Wilhelms-University of Münster, Albert-Schweitzer-Strasse 33, 48129 Münster, Germany; 3 Department of Cellular and Molecular Medicine, Howard Hughes Medical Institute, UCSD School of Medicine, 9500 Gilman Drive, La Jolla, CA 92093-0686, USA

**Keywords:** PLC-*γ*1, EGFR, c-erbB-2, migration, TAT

## Abstract

Due to its pivotal role in the growth factor-mediated tumour cell migration, the adaptor protein phospholipase C-*γ*1 (PLC-*γ*1) is an appropriate target to block ultimately the spreading of EGFR/c-erbB-2-positive tumour cells, thereby minimising metastasis formation. Here, we present an approach to block PLC-*γ*1 activity by using protein-based PLC-*γ*1 inhibitors consisting of PLC-*γ*1 SH2 domains, which were fused to the TAT-transduction domain to ensure a high protein transduction efficiency. Two proteins were generated containing one PLC-*γ*1-SH2-domain (PS1-TAT) or two PLC-*γ*1-SH2 domains (PS2-TAT). PS2-TAT treatment of the EGFR/c-erbB-2-positive cell line MDA-HER2 resulted in a reduction of the EGF-mediated PLC-*γ*1 tyrosine phosphorylation of about 30%, concomitant with a complete abrogation of the EGF-driven calcium influx. In addition to this, long-term PS2-TAT treatment both reduces the EGF-mediated migration of about 75% combined with a markedly decreased time locomotion of single MDA-HER2 cells as well as decreases the proliferation of MDA-HER2 cells by about 50%. Due to its antitumoral capacity on EGFR/c-erbB-2-positive breast cancer cells, we conclude from our results that the protein-based PLC-*γ*1 inhibitor PS2-TAT may be a means for novel adjuvant antitumour strategies to minimise metastasis formation because of the blockade of cell migration and proliferation.

The primary cause of death in cancer is not attributed to the primary tumour growth but rather to the formation of metastases at distant organ sites. A key role in this process is the ability of single tumour cells or tumour cell clusters to become motile, thereby detaching from the primary tumour and start to migrate through the surrounding extracellular matrix. A decisive step in the initiation of migration of epidermal growth factor receptor (EGFR)/c-erbB-2-positive breast cancer cells has been attributed to the specific phosphorylation of tyrosine residue 1248 of c-erbB-2 via EGFR, thereby modulating the time course of the adaptor protein phospholipase C-*γ*1 (PLC-*γ*1) (Dittmar *et al*, 2002). Epidermal growth factor receptor and c-erbB-2 belong to the erbB receptor family and they are activated via ligand-induced homodimerisation as well as heterodimerisation (Alroy and Yarden, 1997), whereby c-erbB-2 is the preferred heterodimerisation partner of all erbB receptors (Graus-Porta *et al*, 1997). Epidermal growth factor (EGF)-induced PLC-*γ*1 activation contributes to the engagement of actin-modifying enzymes including gelsolin (Chen *et al*, 1996), resulting in the rearrangement of the actin cytoskeleton, thereby promoting cell migration by increasing the fraction of cells in a motility-permissive morphology (Wells *et al*, 1999). The relationship between EGFR/c-erbB-2 heterodimer signalling and gelsolin activation in breast cancer cells has been called the ‘master switch’ hypothesis (Brandt *et al*, 1999; Thor *et al*, 2001). Epidermal growth factor receptor/c-erbB-2 heterodimers are constitutively formed on breast cancer cells, thereby determining a motogenic phenotype (Brandt *et al*, 1999) through the above-mentioned mechanism (Dittmar *et al*, 2002). In addition to its participation in overall cell migration, there is evidence that the directionality of cell migration is also PLC-*γ*1 regulated. This is regulated via binding of PLC-*γ*1 to phosphatidylinositol-3,4,5-trisphosphate (PIP_3_) (Falasca *et al*, 1998), which in turn is the product of phosphoinositide 3-kinase (PI-3K) that translocates due to an EGF-dependent mechanism at the leading edge of the cell (Piccolo *et al*, 2002).

Inhibition of PLC-*γ*1 activity due to the expression of a dominant-negative PLCz molecule or the pharmacological PLC-*γ*1 inhibitor U73122 resulted in a reduction of the *in vitro* invasiveness of breast and prostate tumour cells (Kassis *et al*, 1999). In addition to this, it was recently shown that the EGF-induced cell migration of EGFR/c-erbB-2-positive breast cancer cells, but not of solely EGFR-positive breast cancer cells, could be blocked with moderate concentrations of the PLC-*γ*1 inhibitor U73122 (Dittmar *et al*, 2002). Thus, inhibition of PLC-*γ*1 activity is an appropriate way to block EGF-driven tumour cell migration and invasiveness.

However, although Turner *et al* (1996) reported that intraperitoneal administration of U73122 blocks the invasiveness of prostate cancer cells in a mouse model, the *in vivo* usage of this pharmacological inhibitor has to be balanced critically since U73122 has to be dissolved in organic solvents and tends to precipitate when transferred to aqueous solutions, thereby losing its inhibitory activity. In addition to this, it was shown by Rossler *et al* (1998) that U73122 also blocks PLC-*β* activity. As a consequence of this, the possible side effects of both U73122 as well as the organic solvent could not be excluded. In the present study, we designed an approach to block the EGF-induced cell migration of EGFR/c-erbB-2-positive breast cancer cells by using protein-based PLC-*γ*1 inhibitors. These inhibitors consist of either one (PS1-TAT) or two (PS2-TAT) PLC-*γ*1 SH2 domains that were fused to the TAT leader motif of the HIV-TAT protein (Nagahara *et al*, 1998) to ensure a high transduction efficiency. In this study, we will provide evidence of the feasibility of protein-based PLC-*γ*1 inhibitors as potent antitumour agents due to their ability to block PLC-*γ*1 activity efficiently, thereby inhibiting the proliferation as well as the EGF-induced migration of EGFR/c-erbB-2-positive breast cancer cells.

## MATERIAL AND METHODS

### Cells and culture conditions

The breast adenoma carcinoma cell line MDA-MB-468-HER2 (MDA-HER2; EGFR and c-erbB-2 positive) (Dittmar *et al*, 2002) was maintained in Leibowitz L-15 media (PAA, Linz, Austria) supplemented with 10% heat-inactivated foetal calf serum (PAA), 10 U ml^−1^ penicillin, 10 mg ml^−1^ streptomycin and 400 *μ*g ml^−1^ G418 (Merck BioSciences GmbH, Schwalbach, Germany) in 37°C humidified atmosphere without CO_2_ supplementation.

### Cloning

**Figure 1:**
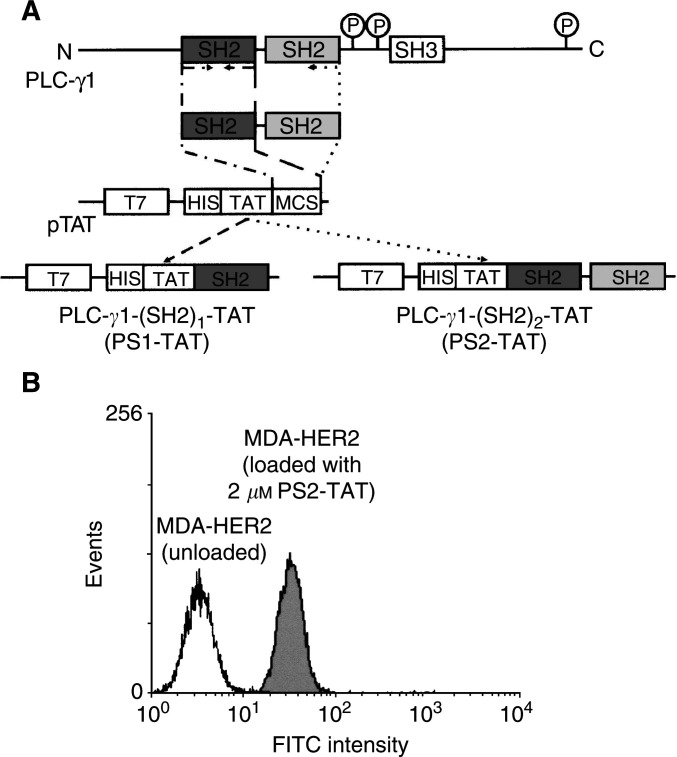
Schematic cloning of PS1/2-TAT proteins and transduction efficiency. (**A**) The PLC-*γ*1-(SH2)_1_ and the PLC-*γ*1-(SH2)_2_ inserts were amplified by RT–PCR and cloned in-frame into the pTAT expression vector. The yielded expression vectors coding for the particular protein were entitled PS1-TAT and PS2-TAT. (**B**) The uptake of the recombinant PS1/2-TAT fusion proteins into MDA-HER2 cells was analysed by flow cytometry using FITC-labelled PS1/2-TAT fusion proteins. Both PS1-TAT as well as PS2-TAT rapidly transduced into 100% of MDA-HER2 cells, achieving maximum intracellular concentration within 5 min. This figure shows a representative histogram plot of MDA-HER2 cells positively transduced with 2 *μ*M PS2-TAT. Untreated MDA-HER2 cells served as a control.

The PLC*γ*1-(SH2)_1_ and PLC*γ*1-(SH2)_2_ DNA sequences were obtained by RT–PCR of MDA-NEO mRNA (forward primer (FP): 5′-CTA GGT ACC AAG GAG GTC AGC AGC AGC-3′, PLC-*γ*1-(SH2): reverse primer (RP): 5′-GAT CGA ATT CAT CTC AAA CTC ATT ACA GCG-3′; PLC-*γ*1-(SH2)_2_: RP: 5′-CTA GAC AAG CTT CAC TCC TCG TTG ATG GGA TAG CG-3′) (all primers: MWG-Biotech AG, Ebersberg, Germany) and were cloned in-frame into the bacterial pTAT expression vector (Nagahara *et al*, 1998) to produce TAT fusion proteins (Figure 1A

). The obtained plasmids pPLC*γ*1-(SH2)_1_-TAT (PS1-TAT) and pPLC*γ*1-(SH2)_2_-TAT (PS2-TAT), respectively, were transformed into the *Escherichia coli* strain XL-1-Blue and subsequently screened by restriction analysis. Positive recombinants were sequenced additionally prior to transformation into the *E. coli* strain BL21(DE3)pLysS. Bacterial cultures were grown overnight and protein expression was induced by IPTG treatment for 4–6 h followed by sonication in buffer Z (20 mM HEPES (pH 8.0), 8 M urea, 100 mM NaCl) containing 20 mM imidazole. The His-tagged fusion proteins were purified using Ni-NTA-agarose (Qiagen, Hilden, Germany) as described (Nagahara *et al*, 1998). After the elution step, the protein solution was dialysed against PBS using the Slide-A-Lyser dialysis cassette (Pierce Chemical Co., Rockford, IL, USA). Subsequently, the protein solution was concentrated in Centricon tubes (Schleicher & Schuell, Dassel, Germany) and frozen in 10% glycerol at −80°C.

### Determination of protein transduction efficiency

Dose- and time-dependent uptake of PS1/2-TAT proteins was determined by flow cytometry (FACSCalibur, Becton Dickenson, Heidelberg, Germany) using FITC-labelled PS1/2-TAT proteins. Therefore, PS1/2-TAT fusion proteins were labelled with the dye Fluorescein by incubating 10 *μ*g FITC in 100 mM NaCO_3_ (pH 9) with 100 *μ*g of protein for 2 h at room temperature in the dark. Unbound FITC was removed by dialysing the labelled protein against PBS. Protein transduction was performed by simply adding the PS1/2-TAT fusion proteins to the MDA-HER2 cell (6 × 10^4^) suspension.

### Calcium measurements

For the investigation of intracellular calcium changes by treatment with various concentrations of PS1/2-TAT, MDA-HER2 cells (6 × 10^4^) were preloaded with the calcium-sensitive fluorescent dye Fluo3 (Molecular Probes, Leiden, The Netherlands) as previously described with minor modifications (Lang *et al*, 2002), and the measurement was started immediately. The value of the mean Fluo3 fluorescence intensity (MFI) was noted every 10 s for a period of 2 min. The values were calculated relative to the initial value *t*_0_ that was set to 1.

### Immunoprecipitation and Western blot

The analysis of PLC-*γ*1 tyrosine phosphorylation by Western Blot was performed as previously described (Dittmar *et al*, 2002) with minor modifications. MDA-HER2 cells (4 × 10^6^) were pretreated with PS1/2-TAT (133 *μ*M, 30 min) prior to EGF treatment (100 ng ml^−1^, 2 min). Phospholipase C-*γ*1 immunoprecipitation and detection was performed using the specific PLC-*γ*1 antibody (clone E12; Santa Cruz, Santa Cruz, CA, USA). Phospholipase C-*γ*1 tyrosine phosphorylation was detected using affinity-purified rabbit polyclonal antibody PLC-*γ*1(pY783) (BioSource, Solingen, Germany).

### Cell migration analysis

The investigation of cell migration using a three-dimensional collagen lattice assay combined with computer-assisted cell tracking was performed as previously described (Dittmar *et al*, 2002). In brief, liquid collagen solution was mixed with 10 × MEM, sodium bicarbonate, MDA-HER2 cells (6 × 10^4^) and the appropriate substances to be tested. Cell migration within the three-dimensional collagen lattices was recorded for at least 12 h by time-lapse video microscopy and subsequently analysed by computer-assisted cell tracking. For data analysis, 30 cells of each sample were randomly selected and two-dimensional projections of the paths were digitised as *x*/*y* coordinates in 20 min intervals.

### XTT proliferation assay

MDA-HER2 proliferation within the presence of PS1/2-TAT proteins was performed as previously described with minor modifications (Dittmar *et al*, 2000). In short, MDA-HER2 cells (1 × 10^3^) were seeded in quadruplicates in a 96-well microtitre plate and were permanently treated with PS1/2-TAT (2 *μ*M). The medium containing the appropriate amounts of PS1/2-TAT was replaced every 48 h. Plates were analysed on days 0, 4 and 7 with XTT reagent, according to the manufacturer's instruction using a microplate reader.

### Statistical analysis

Statistical analysis was performed using the two-tailed unpaired Student's *t-*test (three independent experiments).

## RESULTS

### MDA-HER2. cells are successfully transduced with PLC-*γ*1-SH2-TAT fusion proteins

Two recombinant PLC-*γ*1-(SH2)_1/2_-TAT fusion proteins were generated in this study by cloning RT–PCR-amplified PLC-*γ*1-SH2 DNA sequences into the pTAT expression vector (Figure 1A). The PLC-*γ*1-(SH2)_2_-TAT fusion protein (PS2-TAT) contains both SH2 domains of PLC-*γ*1, whereas the PLC-*γ*1-(SH2)_1_-TAT fusion protein (PS1-TAT) was generated by inserting only the N-terminal SH2 domain of PLC-*γ*1 (Figure 1A) into the pTAT expression vector. For our studies, we used the MDA-HER2 cell line that is double positive for EGFR and c-erbB-2 expression (Brandt *et al*, 1999; Dittmar *et al*, 2002). The uptake of recombinant PS1/2-TAT fusion proteins into MDA-HER2 cells was analysed by flow cytometry using FITC-labelled recombinant PS1/2-TAT fusion proteins. Both PS1-TAT as well as PS2-TAT rapidly transduced into 100% of MDA-HER2 cells, achieving maximum intracellular concentration within 5 min. Figure 1b shows a representative result of MDA-HER2 cells positively transduced with 2 *μ*M PS2-TAT. In order to ensure that all transduced proteins have refolded correctly, the incubation period was extended to 30 min prior to the start of the desired experiment.

### PS2-TAT but not PS1-TAT blocks the EGF-induced activation of the PLC-*γ*1 signal transduction pathway in MDA-HER2 cells

**Figure 2:**
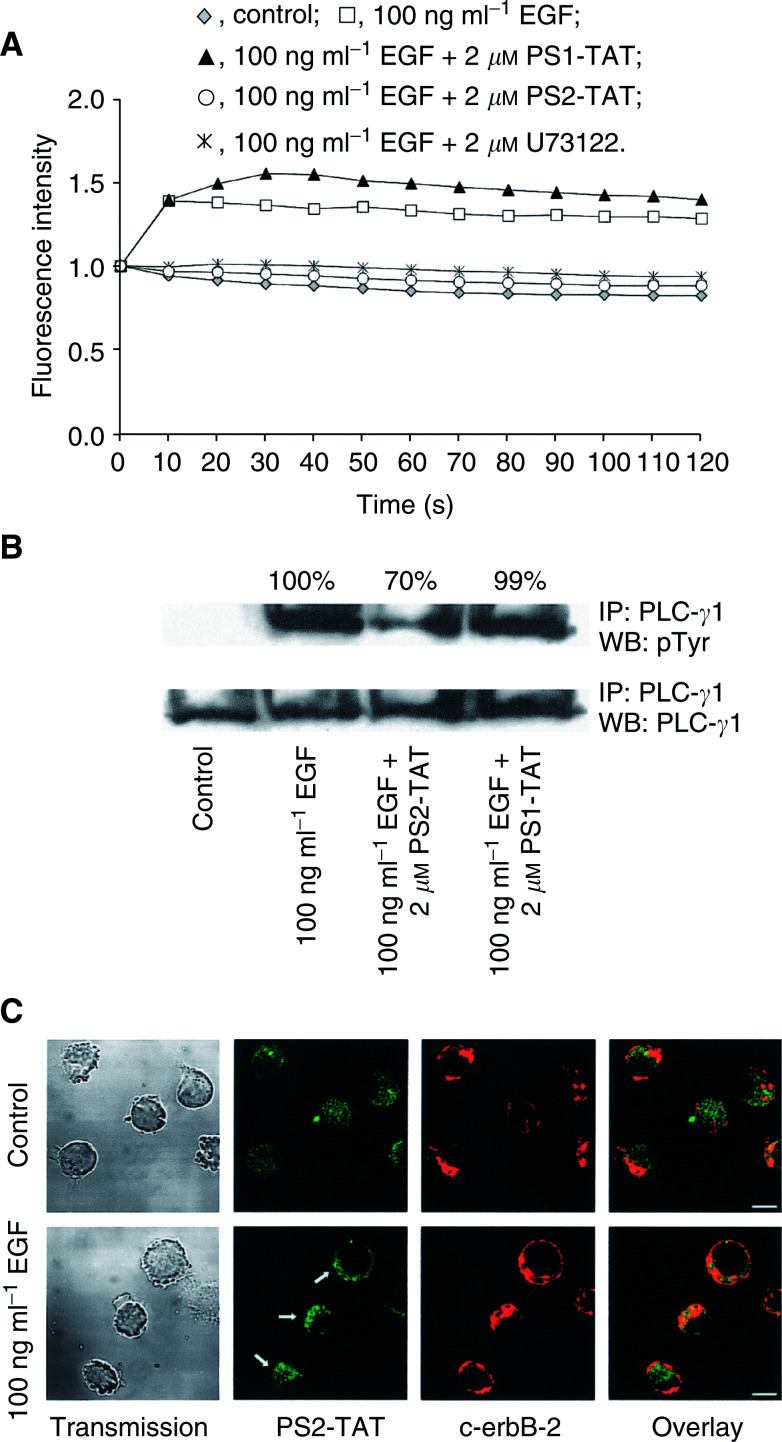
The EGF-induced PLC-*γ*1 activation is blocked by PS2-TAT. (**A**) The EGF-induced calcium influx was used as a read-out to study the inhibitory capacities of the PS1/2-TAT proteins. Treatment of MDA-HER2 cells with PS2-TAT (2 *μ*M) but not PS1-TAT (2 mM) resulted in a complete blockade of the EGF (100 ng ml^−1^)-induced calcium influx. U73122 (2 *μ*M) was used as a control. (**B**) Western Blot analysis was performed to study the mode of action as to how PS2-TAT blocks PLC-*γ*1 activation. Owing to the higher number of cells that were used for these experiments, the amount of PS1/2-TAT was increased accordingly to 133 *μ*M. PS2-TAT treatment resulted in a markedly reduced PLC-*γ*1 tyrosine phosphorylation in the MDA-HER2 cell line after 2 min of EGF (100 ng ml^−1^) stimulation. PS1-TAT, did not interfere with PLC-*γ*1 tyrosine phosphorylation in MDA-HER2 cells. (**C**) Confocal laser scanning microscope analysis revealed a translocation of PS2-TAT (2 *μ*M) proteins towards the plasma membrane of MDA-HER2 cells after 2 min of EGF (100 ng ml^−1^) stimulation, where it is found to be colocalised with the constitutively formed EGFR/c-erbB-2 heterodimer (indicated by c-erbB-2 receptor staining). Bar=10 *μ*m.

In order to investigate whether the recombinant PS1/2-TAT proteins could inhibit PLC-*γ*1 activation, the EGF-driven calcium influx was used as a read-out since the influx of calcium is closely related to PLC-*γ*1 activation (Dittmar *et al*, 2002). The EGF-induced calcium influx of MDA-HER2 cells was analysed by flow cytometry by preloading the cells with the calcium-sensitive fluorescent dye FLUO-3 prior to PS1/2-TAT (2 *μ*M) treatment and EGF (100 ng ml^−1^) stimulation. Stimulation of MDA-HER2 cells with 100 ng ml^−1^ EGF resulted in a moderately increased fluorescence intensity of about 54±4% (due to low basal intracellular calcium levels in this cell line), which was completely abrogated by PS2-TAT (Figure 2A

). Similar results were obtained with the PLC-*γ*1 inhibitor U73122 (2 *μ*M) that served as a control. In contrast to this, PS1-TAT showed no inhibitory capacity (Figure 2a). In order to investigate the mode of action as to how PS2-TAT blocks PLC-*γ*1 activity in MDA-HER2 cells, we determined the grade of PLC-*γ*1 tyrosine phosphorylation by Western blot analysis. Owing to the higher number of cells that were used for Western blot analysis, the amount of PS1/2-TAT was increased accordingly from 2 *μ*M (6 × 10^4^) to 133 *μ*M (4 × 10^6^). MDA-HER2 cells were stimulated with 100 ng ml^−1^ EGF for 2 min since it was shown that PLC-*γ*1 tyrosine phosphorylation is highest after this incubation period (Dittmar *et al*, 2002). The results we obtained were congruent with the calcium influx data, indicating that 2 *μ*M PS2-TAT but not 2 *μ*M PS1-TAT blocks the EGF-induced PLC-*γ*1 tyrosine phosphorylation in MDA-HER2 cells (Figure 2B). We estimated a PS2-TAT-dependent reduction of PLC-*γ*1 tyrosine phosphorylation of about 30%. No differences in PLC-*γ*1 tyrosine phosphorylation were observed in PS1-TAT-treated MDA-HER2 cells as compared to EGF-treated MDA-HER2 cells. Using confocal laser scanning microscopy, we observed that EGF (100 ng ml^−1^) stimulation of PS2-TAT (2 *μ*M)-loaded MDA-HER2 cells resulted in a translocation of the PS2-TAT protein towards the plasma membrane. Costaining of c-erbB-2, which is part of the constitutively formed EGFR/c-erbB-2 heterodimer, revealed a colocalisation of PS2-TAT and the erbB receptor heterodimer (Figure 2C, lower row, white arrows). In untreated MDA-HER2 cells, PS2-TAT is equally distributed within the cytoplasm (Figure 2C, upper row).

### PS2-TAT blocks the EGF-induced migration of EGFR/c-erbB-2-positive MDA-HER2 cells

**Figure 3:**
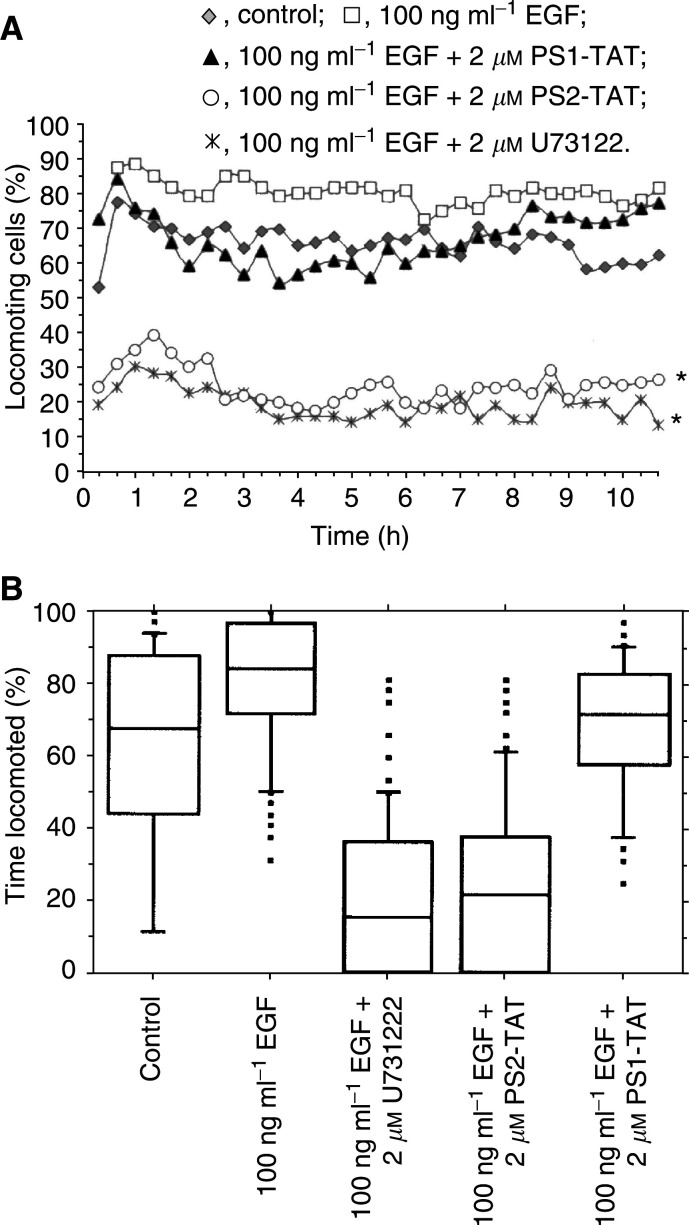
The EGF-induced migration of MDA-HER2 cells is permanently blocked by PS2-TAT. Cell migration was analysed by using the three- dimensional collagen matrix assay combined with computer-assisted cell tracking. (**A**) The mean locomotory activity of EGF (100 ng ml^−1^)-treated MDA-HER2 cells was significantly (^*^=*P*<0.0001) reduced by about 75% by PS2-TAT (2 *μ*M) treatment. Similar results were achieved by U73122 (2 *μ*M) that was used as a control. In contrast to this, PS1-TAT (2 *μ*M) showed a rather marginal inhibition of the EGF (100 ng ml^−1^)-induced migration of MDA-HER2 cells. The graph represents the mean locomotory activity of treated and untreated MDA-HER2 cells of four independent experiments (120 cells). (**B**) The Box-Plot diagram represents the time locomotion of single MDA-HER2 cells. PS2-TAT (2 *μ*M) but not PS1-TAT (2 *μ*M) treatment resulted in a markedly reduced time locomotion of EGF (100 ng ml^−1^)-treated MDA-HER2 cells. U73122 (2 *μ*M) treatment yielded comparable results. Results were obtained from four independent experiments (120 cells).

Since PLC-*γ*1 is a key player in the EGF-mediated induction of tumour cell migration, we next analysed the migratory behaviour of PS1/2-TAT-treated MDA-HER2 cells by using the three-dimensional collagen matrix migration assay combined with computer-assisted cell tracking. Untreated MDA-HER2 cells displayed a mean spontaneous locomotory activity of about 66.0±4.9% that was significantly increased by EGF (100 ng ml^−1^) treatment to 80.5±3.4% (*P*<0.0001, Figure 3a

) and that is congruent to previously published data (Dittmar *et al*, 2002). The EGF (100 ng ml^−1^)-induced migration of MDA-HER2 cells was significantly and permanently blocked by PS2-TAT (2 *μ*M) treatment of about 75% to solely 24.8±5.3% (*P*<0.0001; Figure 3a). Similar results were obtained with the PLC-*γ*1 inhibitor U73122 (2 *μ*M) that was used as a control. Here, the locomotory activity was reduced to 19.4±4.4% (*P*<0.0001; Figure 3a). These results clearly indicate that the observed reduced migration of PS2-TAT-treated MDA-HER2 cell is attributed to PS2-TAT-mediated PLC-*γ*1 inhibition. In contrast to PS2-TAT, the inhibitory capacity of PS1-TAT was rather moderate (Figure 3a). In the first 7–8 h, the locomotory activity of EGF- and PS1-TAT-treated MDA-HER2 cells was solely reduced to the control level of untreated MDA-HER2 cells. After that time, the locomotory activity increased steadily, finally reaching the locomotory activity level of EGF-treated MDA-HER2 cells. In mean, we estimated a locomotory activity of PS1-TAT- and EGF-treated MDA-HER2 cells of about 66.9±7.5%. In addition to the determination of the average locomotory activity of the MDA-HER2 cell population, we also analysed the time locomotion of single MDA-HER cells, which represents the time of how long a single cell has migrated during the whole observation time. This is a crucial parameter that has to be determined when investigating cell migration since changes in the locomotory activity of a cell population in response to a stimulus or inhibitor could be attributed either to a change in the total number of migrating cells or to a change in the total time of locomotion of single cells or to a combination of both modi operandi. The results of the time locomotion are displayed in a Box-Plot diagram, whereby the Box-Plot indicates the distribution of the single values with 50% of all values lying within the Box. It can be clearly deduced from Figure 3b that the EGF (100 ng ml^−1^)-induced time locomotion of MDA-HER2 cells is markedly decreased by 2 *μ*M PS2-TAT treatment. While EGF-stimulated MDA-HER2 cells locomoted 72–97% of the observed time period, PS2-TAT treatment reduced the locomoting time to 0–38%. Similar results were obtained using the PLC-*γ*1 inhibitor U73122 (Figure 3B). Here, we observed a time locomotion of 0–36% and the total number of EGF-induced MDA-HER2 cells was decreased to 50.0±15.3%. In agreement with the slightly reduced EGF-induced migration of PS1-TAT-treated MDA-HER2 cells, we determined a weakly decreased locomoting time of 58–82% (Figure 3B).

### PS2-TAT decreases the proliferation rate of MDA-HER2 cells

**Figure 4:**
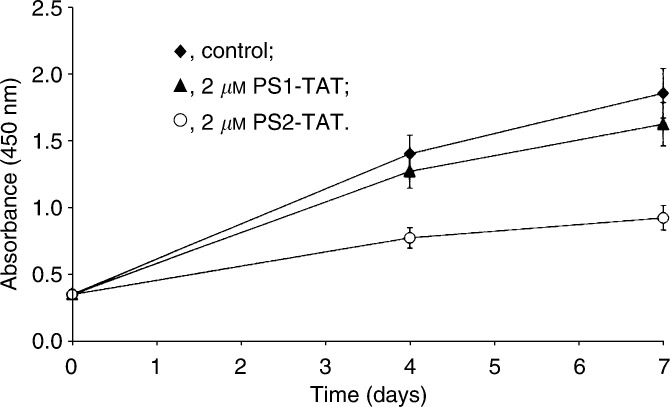
PS2-TAT long-term treatment decreases the proliferation of MDA-HER2 cells. MDA-HER2 cells were permanently incubated within the presence of 2 *μ*M PS1/2-TAT. Cell proliferation was measured by XTT assay on days 0, 4 and 7. Compared to control cells, the proliferation of PS2-TAT (2 *μ*M)-treated MDA-HER2 cells was reduced by about 50%. PS1-TAT (2 *μ*M) treatment showed a rather marginal effect. Each value is the mean±s.e. of quadruplicate data.

In addition to the cell migration data indicating a long-term inhibitory capacity of PS2-TAT, we performed an XTT proliferation assay to investigate whether long-term PS2-TAT (2 *μ*M) treatment of MDA-HER2 cells also influenced the proliferation rate of this cells. Owing to the above-mentioned limitations of U73122 and its worse solubility in aqueous solutions, the U73122-dependent cell proliferation of MDA-HER2 cells was not determined. The results are summarised in Figure 4

and it could be clearly deduced from the graph that long-term PS2-TAT treatment resulted in a decreased proliferation rate of MDA-HER2 cells. Compared to the proliferation rate of control cells, the growth of PS2-TAT-treated MDA-HER2 cells was reduced by about 55% after 4 days and 50% after 7 days, respectively. In accordance with the weakly influenced EGF-induced migration of MDA-HER2 cells, we observed a slightly reduced proliferation of PS1-TAT-treated MDA-HER2 cells. Here, we estimated a proliferation rate of about 91% after 4 days and 88% after 7 days as compared to the proliferation of control cells.

## DISCUSSION

In the present study, we generated protein-based PLC-*γ*1 inhibitors consisting of PLC-*γ*1-SH2 domains fused to the TAT leader sequence as highly specific inhibitors to block PLC-*γ*1 tyrosine phosphorylation, thereby inhibiting the EGF-induced migration of EGFR/c-erbB-2-positive breast cancer cells. The adaptor protein PLC-*γ*1 was chosen as a target due to its pivotal role in the EGF-dependent migration and invasiveness of tumour cells mediated by EGFR and c-erbB-2 signalling. Epidermal growth factor-induced PLC-*γ*1 activation contributes to the engagement of actin-modifying enzymes including gelsolin (Chen *et al*, 1996), resulting in the rearrangement of the actin cytoskeleton, thereby promoting cell migration by increasing the fraction of cells in a motility-permissive morphology (Wells *et al*, 1999). In addition to this, it was recently demonstrated by Dittmar *et al* (2002) that the induction and maintenance of the EGF-mediated cell migration of EGFR/c-erbB-2-positive breast cancer cells is driven by the c-erbB-2-dependent EGFR/c-erbB-2 heterodimer signalling, thereby modulating the PLC-*γ*1 kinetics.

Two protein-based PLC-*γ*1 inhibitors were generated consisting of either one (PS1-TAT) or two (PS2-TAT) PLC-*γ*1-SH2 domains, whereby the PS1-TAT protein contains the N-terminal SH2-domain of PLC-*γ*1. The PLC-*γ*1 N-terminal SH2 domain was chosen since it binds to the c-erbB-2 tyrosine residue 1248, which in turn plays a key role in PLC-*γ*1 activation (Dittmar *et al*, 2002). The treatment of MDA-HER2 cells with the PS2-TAT protein resulted in a reduced PLC-*γ*1 tyrosine phosphorylation, a markedly decreased EGF-induced cell migration as well as a reduced proliferation of MDA-HER2 cells. The confocal laser scanning microscopy results show that activation of the EGFR/c-erbB-2 heterodimer results in a translocation of the PS2-TAT to the plasma membrane where it is found to be colocalised with the EGFR/c-erbB-2 heterodimer complex. Together with the Western blot data showing a reduction in PLC-*γ*1 tyrosine phosphorylation, we conclude that the PS2-TAT protein most likely competes with the native PLC-*γ*1 protein for binding to the activated EGFR/c-erbB-2 heterodimer, thereby inhibiting PLC-*γ*1 activation.

However, it remains unclear as to why PS1-TAT showed no or solely a weak PLC-*γ*1 inhibition, although PS1-TAT should bind to c-erbB-2 tyrosine residue 1248 for which a pivotal role in PLC-*γ*1 activation has been described (Dittmar *et al*, 2002). Since we observed a slightly decreased PLC-*γ*1 tyrosine phosphorylation in the solely EGFR-positive MDA-MB-468-NEO cell line (data not shown), we presume that the underlying reasons for the noninhibitory capacity of PS1-TAT are likely attributed to the different binding affinities of the PS1-TAT protein to the appropriate activated erbB receptor. The finding that both PLC-*γ*1 SH2 domains are necessary for a complete blockade of PLC-*γ*1 activation, thereby inhibiting cell migration, are consistent with the findings of Kassis *et al* (1999), who reported that the expression of a dominant-negative PLCz domain, consisting of both SH2, the SH3 and an inhibitory (I) domain, resulted in a reduction of the invasiveness of the EGFR/c-erbB-2-positive cell line MDA-MB-362. However, protein expression in eukaryotic cells requires an efficient DNA transfection and protein expression system. In contrast to this, the chosen TAT-transduction system overcomes the limitations that could occur in case of DNA transfection such as low transfection efficiency. The TAT-transduction technique allows a fast and concentration-dependent delivery of TAT fusion proteins into nearly 100% of cells, where they remain for 48 h until their degradation (Schwarze and Dowdy, 2000; Abu-Amer *et al*, 2001). Using the TAT protein transduction technology, protein purification can be performed regardless of renaturation or folding since misfolding enhances the uptake of the protein into the cells where it gets folded correctly (Nagahara *et al*, 1998). In addition to this, this approach also overcomes the limitations that could occur in case of U73122 usage since this inhibitor has to be dissolved in organic solvents and tend to precipitate when transferred to aqueous solutions, thereby losing its inhibitory capacity. Furthermore, U73122 also blocks PLC-*β* activity (Rossler *et al*, 1998) and therefore possible side effects of both U73122 as well as the organic solvent could not be excluded.

Long-term PS2-TAT treatment of MDA-HER2 cells resulted in a markedly decreased EGF-driven migration as well as in a reduced proliferation rate of these cells. These results clearly indicate that PS2-TAT has a long-term inhibitory capacity, which in turn is a prerequisite for a possible therapeutic relevance. Several *in vivo* studies using TAT fusion proteins as active compounds have already been described. Harada *et al* have demonstrated successfully the antitumour effect of a TAT-oxygen-dependent degradation-caspase-3 fusion protein, which is specifically stabilised and activated in hypoxic tumour cells. Intraperitoneal injection of this TAT fusion protein into a tumour-bearing mouse resulted in a reduction of the tumour size (Harada *et al*, 2002). Besides the usage of TAT fusion proteins as anticancer drugs, a TAT-bcl-xl fusion protein was successfully used as a neuroprotective substance in order to prevent neuronal death after ischaemic brain injury (Cao *et al*, 2002) or transient focal ischaemia (Kilic *et al*, 2002) in mice.

In conclusion, our data show that protein-based PLC-*γ*1 inhibitors are not only potent PLC-*γ*1 inhibitors but also possess antitumour effects on EGFR/c-erbB-2-positive breast cancer cells due to the inhibition of both proliferation and migration.
